# Novel Acoustic Technology for Studying Free-Ranging Shark Social Behaviour by Recording Individuals' Interactions

**DOI:** 10.1371/journal.pone.0009324

**Published:** 2010-02-19

**Authors:** Tristan L. Guttridge, Samuel H. Gruber, Jens Krause, David W. Sims

**Affiliations:** 1 Institute for Integrative and Comparative Biology, University of Leeds, Leeds, United Kingdom; 2 Bimini Biological Field Station, South Bimini, Bahamas; 3 Division of Marine Biology and Fisheries, Rosenstiel School of Marine and Atmospheric Science, Miami, Florida, United States of America; 4 Department of Biology and Ecology of Fishes, Leibniz-Institute of Freshwater Ecology and Inland Fisheries, Berlin, Germany; 5 The Laboratory, Marine Biological Association of the United Kingdom, Citadel Hill, Plymouth, United Kingdom; 6 Marine Biology and Ecology Research Centre, Marine Institute, School of Marine Sciences and Engineering, University of Plymouth, Drake Circus, Plymouth, United Kingdom; University of Canterbury, New Zealand

## Abstract

Group behaviours are widespread among fish but comparatively little is known about the interactions between free-ranging individuals and how these might change across different spatio-temporal scales. This is largely due to the difficulty of observing wild fish groups directly underwater over long enough time periods to quantify group structure and individual associations. Here we describe the use of a novel technology, an animal-borne acoustic proximity receiver that records close-spatial associations between free-ranging fish by detection of acoustic signals emitted from transmitters on other individuals. Validation trials, held within enclosures in the natural environment, on juvenile lemon sharks *Negaprion brevirostris* fitted with external receivers and transmitters, showed receivers logged interactions between individuals regularly when sharks were within 4 m (∼4 body lengths) of each other, but rarely when at 10 m distance. A field trial lasting 17 days with 5 juvenile lemon sharks implanted with proximity receivers showed one receiver successfully recorded association data, demonstrating this shark associated with 9 other juvenile lemon sharks on 128 occasions. This study describes the use of acoustic underwater proximity receivers to quantify interactions among wild sharks, setting the scene for new advances in understanding the social behaviours of marine animals.

## Introduction

Many animals across a diversity of taxa are recognized to form groups [1]. The durations of such groups can differ greatly; some may last for years, others just minutes or even a few seconds [2]. Groups provide animals with the opportunity to interact with other individuals and can vary in their composition, based on numerous phenotypic, physiological and ecological factors [3]–[4]. For large marine vertebrates, such as sharks, the formation of groups is recognised in many species and is thought to provide distinct behavioural advantages, such as in foraging, reproduction or by reducing an individual's risk to predation [5]–[7]. Most studies of grouping behaviour in sharks however, have been either observations on captive sharks, anecdotal or inferred through fishery capture records, or from conventional telemetry [8]–[11]. Shark behaviour is notoriously difficult to study especially when attempting to obtain accurate information on group composition [12], [13]. For this reason, little systematic data is available on the structure and size of these groups, or indeed the timing and frequency of interactions between individuals within them.

In recent years, researchers have become increasingly reliant on remote devices to address a wide range of science and management questions, in a variety of species, including; marine mammals, turtles, teleosts, chondrichthyans, crustaceans and cephalopods [14]–[16]. Tools such as biotelemetry (radio and acoustic telemetry) and biologging (archival logger) devices offer a sophisticated means of evaluating the behaviour, spatial ecology, energetics, and physiology of free-living animals in their natural environment [15]. However, the extent to which these can be used to investigate interactions within and between large aquatic animals, such as sharks, remains relatively unexplored [17]–[19]. Simultaneous detections on submersible underwater receivers (SUR) or manual tracks of multiple sharks are possible, but telemetry spatial error typically negates the possibility of determining the proximity of individuals with a high degree of accuracy [7], [16]. However, previous research undertaken on white sharks (*Carcharodon carcharias*) used a radio-acoustic positioning system, linked to a base station, to investigate social hunting behaviours [17], [18]. Positions of transmitter-tagged sharks were estimated when pulses arrived at three hydrophones mounted on buoys aligned in a triangular array. Spatial accuracy was determined to be 2 to 10 m within an area of 1 km^2^ providing the resolution to determine inter-individual distances. However, having receivers fixed in location meant that the detectable area was restricted and animals moving out of range could not be included in the analysis. To overcome this problem a very recent study described the use of inter-animal telemetry [19]. Galapagos sharks (*Carcharhinus galapagensis*) were equipped with tags that both transmitted their own code and stored signals (i.e. tag number) from other tagged animals. Field trials determined that the tags were capable of accurately capturing the presence-absence patterns of other tagged sharks. Furthermore, the trials demonstrated that on-shark tags can provide important inter- and intra-specific interaction data among individuals in areas remote from traditional fixed receiver arrays [19]. However, at present with this technology it is not possible to determine the distance between tagged sharks. Sharks can be detected ≤1000 m from each other making it difficult to elucidate information regarding individual interactions, such as social behaviours, predator prey encounters and courtship/mating events.

Similar problems have also been identified for terrestrial animals where direct observations might be impractical due to the elusive or nocturnal nature of a species [20]. To combat these issues novel proximity data loggers that measure the frequency and duration of contacts between individuals have been used to derive estimates of contact rates between individuals (e.g. wild-living bush tail possums, *Trichosurus vulpecula*) and to examine relationships of contact rate with population density and habitat [21]. More recently, their application to badgers (*Meles meles*) and dairy cattle in the UK has enabled the identification of high-risk individuals in the transmission of Bovine Tuberculosis (TB) [22].

In this study, we investigate whether acoustic telemetry can be used to study grouping behaviours in sharks. We assess a novel acoustic-proximity receiver in a series of controlled experiments using captive juvenile lemon sharks *Negaprion brevirostris*, testing the detection range and performance of the devices. Finally, we present a demonstration that proximity receivers deployed on free-ranging lemon sharks yield individual interaction data, indicating their potential use in locating shark aggregation sites and determining associative patterns (for example social interactions based on size or sex) between sharks within a population. We also discuss other uses for this type of technology in shark research, with an obvious extension to its use being other marine animals, and consider further advances and future experiments that may serve to improve the tag's performance and application.

## Materials and Methods

### Ethics Statement

All surgical procedures were conducted in accordance with the animal welfare laws of the country in which they were undertaken.

### Study Site and Species

**Figure 1:**
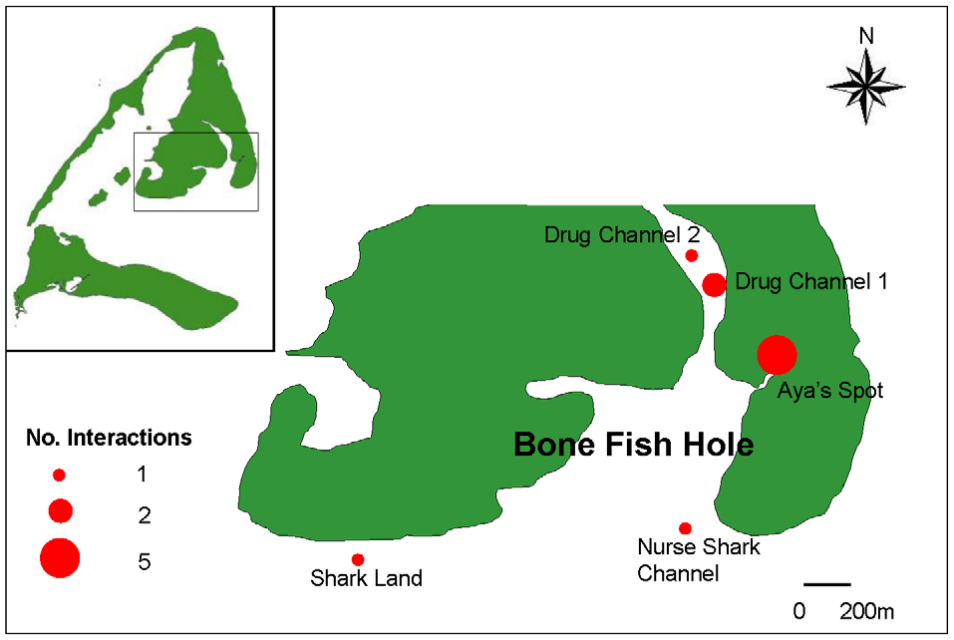
Study and Interaction sites. Map showing study site and locations of lemon shark associations around East Bimini, Bahamas.

This study was conducted in Bimini, Bahamas, a small chain of islands approximately 85 km east of Miami, Florida, U.S.A (Fig. 1). Juvenile lemon sharks were used as test subjects because of their abundance in Bimini, renowned hardiness in captivity, relatively small body size, extensive overlapping of home ranges and known conspecific encounters [23]–[25]. These sharks were also a practical species when conducting the free-ranging trials due to their restricted movement as juveniles, where they spend considerable time in small areas and show high site fidelity [24]–[26] allowing for recapture and retrieval of the acoustic receivers.

### Proximity Receivers

**Figure 2:**
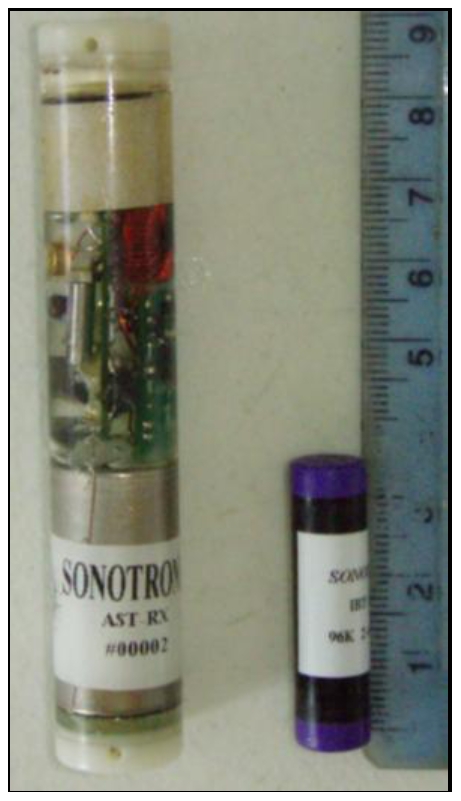
Animal-attached/implanted proximity detector. Example of the ARX acoustic receiver (left) and IBT transmitter (right) used in the study on lemon sharks.

The acoustic receivers used in this study were a prototype system (ARX-RX1, Sonotronics Inc, Tuscan, Arizona, USA) designed to log the date and time of transmitter-tagged individuals moving within the receiver's detection range (Fig. 2). Each ARX device acts as a miniature omni-directional SUR capable of detecting multiple ultrasonic transmitters set to 96 kHz frequency, within a specified distance. The ARX receivers were tested and passed through a series of electrical trials, in the factory, to a sensitivity of −71dbV +/− 1dbV. This gain setting can be altered to achieve greater or lower sensitivity depending on the detection range required in the study. For this study the above ARX sensitivity setting was estimated by Sonotronics (pers. Comm. Marlin Gregor) to detect transmitters within ∼4 m, which is equivalent to ∼4 body lengths of the sharks used in these experiments.

The transmitters used in this study can be detected by the ARX receivers, traditional SUR's and manual tracking receivers. Each acoustic transmitter (IBT, Sonotronics Inc.), as per traditional acoustic tracking technology [16], is assigned an aural code (e.g. 3-3-3). This code takes 20 seconds to pulse at a frequency of 96 kHz. The time intervals (e.g. 910 ms) between each ping of the code are unique to each transmitter, enabling the ARX to discriminate between transmitters. The ARX takes 3 to 4 seconds to identify the broadcasting transmitter's unique time interval, meaning that an individual transmitter can in theory be detected up to 5 times during a 20 second transmission. Each transmitter then stops broadcasting its aural code for 8 seconds, providing an opportunity for ‘competing’ transmitters to be heard and decoded by the ARX. So, for example, during a 60 second period, each transmitter will have two 20- second aural-code broadcasting periods and two 8 second breaks, meaning that the maximum possible number of detections per minute for each transmitter is *ca.* 10. The transmitter will continue this cycle of broadcasting its aural code for 20 seconds and sleeping for 8 seconds until its battery is exhausted (for the IBT tags this is 60 days).

Prior to deployment, the ARX is initiated by placing it on an interface unit that enables an infra-red connection to be established, and that starts an internal clock that counts seconds until stopped. When a transmitter is detected, the time in seconds is logged along with the interval ID (e.g. 910 ms) of the transmitter. This data is then stored in non-volatile memory, within the ARX, and is retrieved through connecting to the interface unit and downloading to a Windows application. The ARX used in this study can differentiate between 50 tags (intervals spaced 10 ms apart), has a battery life of *ca.* 30 days and is capable of keeping records of 106,000 detections, which equates to just over 2 detections per minute. The ARX's memory can be erased for re-use and the unit itself can be set to a ‘sleep’ mode that saves battery life between trials. It is also important to note that both memory capacity and battery life are dependent on ARX size, and hence, the weight-carrying capacity of the species being studied. In this study, the juvenile lemon sharks used were 0.80–0.90 m in total length, weighing 3.0–3.5 kg in air. The ARX unit weighed 31 g in air (16g in sea water); this is less than 1% of the body weight of these sharks, a percentage that has been shown in other studies not to affect shark behaviour [27], [28].

### Experiment 1 – Stationary Test

The first preliminary trial that was conducted was a test to check if the unit worked in a stationary, controlled environment. An ARX receiver and transmitter were attached to separate PVC poles and kept at an in-water height of 0.20 m above a flat, sand seabed. Water depth remained between 0.70 and 0.80 m throughout the treatments. Two treatments were completed: (1) A transmitter was stationed 1 m horizontal distance from the ARX receiver, and (2) 10 m horizontal distance. Both treatments lasted 30 minutes. Based on our previous calculation of a theoretical maximum of 10 detections per minute, an expected number of detections for this time period would be *ca.* 300 detections. The percentage of transmitter signals detected by the receiver was then calculated to give an estimate for the measure of efficiency at the two distances.

### Experiment 2 – On-Shark Trial

A total of 9 juvenile lemon sharks were used in these trials (total length, 0.80 to 0.90 m). All sharks were captured using gillnets, immediately measured and transported to a holding pen, see [29] for details of capture/processing techniques and housing environment. Experiments were conducted in a separate pen, comprised of three compartments, two outer ones (4m×2m) and a central one (4m×10m). This setup was used to test how the ARX receivers performed when fitted to interacting sharks (i.e. two sharks within 4 body lengths or 4m of each other, in one compartment) and also to determine whether detections were recorded when sharks were separated by at least 10 m. Prior to experimentation, 5 sharks were each fitted with an ARX receiver and 4 with an IBT transmitter, through the first dorsal fin. Each shark fitted with an ARX receiver (*N* = 5) completed two treatments with each shark fitted with an IBT tag (*N* = 4). Treatment 1: two sharks were placed together in compartment A or B (one with ARX receiver and one with IBT transmitter); and Treatment 2: individual sharks were placed in separate compartments A and B (one with an ARX receiver and one with a transmitter). Each treatment lasted 10 minutes with sharks given 5 minutes to acclimatise to the compartment and attachment, before trials commenced. An observer recorded the start and end time of each treatment (to the nearest second). The start and end times were compared to detections recorded on each ARX, to determine during which treatments detections were successfully made. During all treatments sharks were observed to swim continuously, indicating that the attachment method did not affect the shark's ability to move around the compartments. When the sharks were together they were observed to interact continuously, performing social behaviours, such as following and circling each other, and so were always <4 m distance from one another. Experiments were performed over a sandy substrate and between 0.70 and 1.00 m water depth. For both of these trials a measure of efficiency (%) was also calculated for the receivers [no. of detections/100 (max no. of detections per minute x trial time)].

### Experiment 3 – Field Trial

Fifteen juvenile lemon sharks were captured using gill and seine nets from Bone Fish Hole nursery area, Bimini, Bahamas (Fig. 1). All were measured and weighed as before, and implanted with a 60 day transmitter (IBT, Sonotronics Inc), using the technique described in [30] before release. These sharks were then monitored for a two week period, through a combination of active and passive telemetry tracking [26], to identify their fidelity to the nursery area. Five sharks showing some site fidelity were then selected for the ARX receiver field trial: all were 0.80 to 0.90 m total length and weighed between 3.0 to 3.5 kg, to ensure the weight of the ARX would not impact on their natural behaviour. These sharks were captured over a period of 3 days and transported in 200L plastic tanks, via boat, to separate semi-captive pens. Sharks were given 24 hours to recover from their capture and were then implanted intraperitoneally with an ARX receiver and transmitter. This method of transmitter attachment is commonly used in studies of shark behaviour and should not alter the detection efficiency of the receiver [7], [16], [26]. Prior to implantation each ARX was programmed to ignore the IBT transmitter that they were paired with, to ensure that the memory would not fill up with the ARX-tagged shark's own acoustic-transmitter tag ID. All transmitters were positioned with the same orientation within the shark's body cavity and dissolvable sutures were used. The sharks were given 7 days to recover from surgery and were fed to satiation on fresh local fish every 2 days. After this period, sutured incisions on sharks were checked to ensure that they were sealed and sharks were released to the wild for two weeks. At the end of this free-ranging test period, four of the five sharks were successfully recaptured over a 4 day period using gillnets and their ARX receiver tags were removed (using non-lethal techniques described in [26]) and data were downloaded.

Proximity does not necessary imply a social interaction. However, longer durations of proximity are likely to be indicative of interactions. To address this problem in our field data, we used the results from experiment 2 to set a criterion for identifying a social interaction between two sharks in the wild. So for our wild results, if ≥2 detections were recorded in a 60 second period we recorded it as a social interaction lasting 60 seconds. If detections continued to be within 60 seconds of each other the time between the first and last detection were estimated to be the interaction duration, until two detections were separated by a time period of >60 seconds. Single records were also identified on multiple occasions and were included as interactions of <30 seconds. These may be better interpreted as passing encounters rather than actual social interactions, which in this study at least were assumed to have greater longevity.

To provide spatial information for the interactions the data retrieved from each seabed-mounted receiver (SUR) was assessed to see if it overlapped by 5 minutes for any detections on the ARX receiver tag. The SURs have a detection range of *ca.* 200 m radius. Previous studies have shown that the average swimming speed of a juvenile lemon shark is about 0.7 m s^−1^ [28], so if a shark was to swim at this speed for 5 minutes then it would swim 210 m. We therefore assumed that the interaction took place either in the SUR range or certainly very close by.

## Results

### Experiment 1 – Stationary Trial

This initial trial showed that the ARX receiver worked well at close range distances (1 m), with 127 detections in a 30 minute period (*ca.* 4 min^−1^) indicating a 42% probability of detection. When the distance was extended to 10 m the ARX receiver detected only 3 time intervals in 30 minutes, with a probability of detection at this distance being 1%.

### Experiment 2 – On-Shark Trial

For treatment 1, when sharks were interacting constantly with each other (within 4 body lengths or <4 m apart), ARX receivers averaged 16.95 detections (±S.D. = 6.63) in 10 min, which is equivalent to a single detection every 40 seconds or a receiver average of 16% probability of detection (Table 1). For treatment 2, when sharks were separated by at least 10 m, all ARX units received ≤2 detections (mean±S.D. = 0.73±0.7) throughout the 10 min trials, equivalent to a receiver average of 0.73% probability of detection (Table 1). Although the number of detections between individual ARX receivers was variable, the different treatments demonstrated that at distances <4 m between receiver and transmitter the number of detections was consistently much higher than at distances of 10 m.

**Table 1:** Validation tests of detection frequency (number of received transmissions) as a function of distance between receivers and transmitters during on-shark trials (ARX receivers, *N* = 5; Trials, *N* = 4).

	Transmitter distance to Shark							
Trial	1	1	2	2	3	3	4	4
ARX	10 m	<4 m	10 m	<4 m	10 m	<4 m	10 m	<4 m
**1**	0	30	0	19	0	3	1	17
**4**	2	23	1	15	0	20	1	13
**5**	0	19	0	13	1	20	1	18
**7**	2	26	1	17	0	8	1	15
**8**	0	21	0	14	1	23	2	5

### Experiment 3 – Field Trial

**Figure 3:**
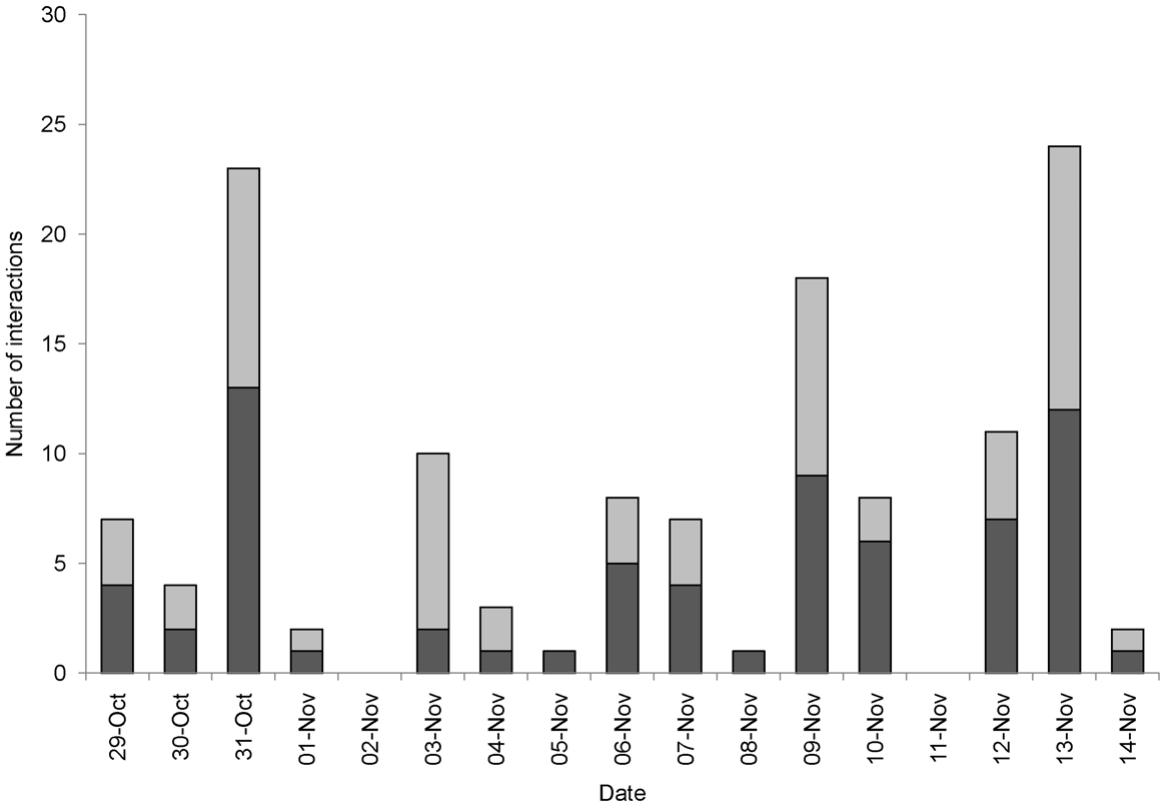
Shark interactions. Daily total number of interactions for shark 365, recorded from ARX receiver #5. Dark grey bars represent interactions lasting <30 seconds, and light grey bars denote interactions lasting ≥60 seconds.

**Figure 4:**
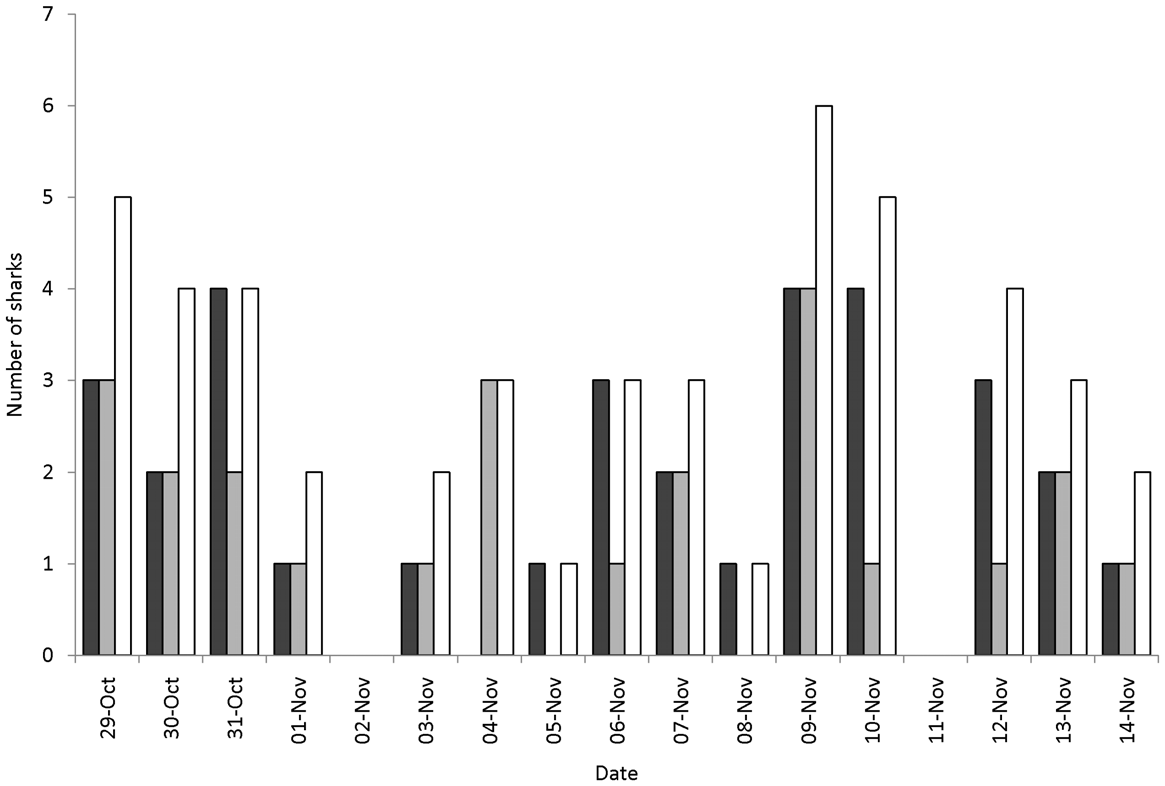
Social partners. Daily number of different sharks that shark 365 interacted with that were recorded by ARX receiver #5. Dark grey bars represent the number of sharks interacted with for <30 seconds, light grey bars are the number of sharks interacted with for ≥60 seconds, and white bars show the total number of sharks interacted with on each day.

**Figure 5:**
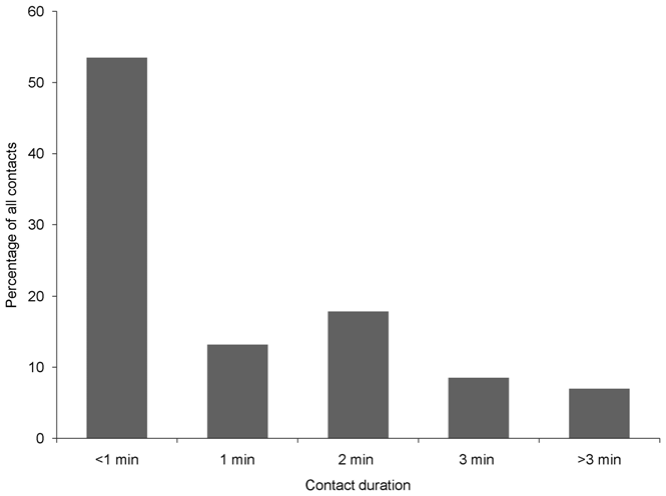
Duration of interactions. Percentage frequency distribution of the length of time shark 365 interacted with other juvenile lemon sharks in the Bone Fish Hole nursery area.

One of the 5 ARX receivers deployed on juvenile lemon sharks was recovered with recorded data. The three retrieved receivers that did not work appeared to have malfunctioned due to programming error, such that instead of just ignoring the transmitter paired with the receiver on the same shark, all transmitters were ignored. The ARX receiver that functioned correctly (ARX #5, shark 365) recorded a total of 315 detections, over a period of 17 days in the Bone Fish Hole nursery area. These detections were from a total of 9 other juvenile lemon sharks, with detections on every day apart from two, and with up to 11 interactions per day of duration ≥60 seconds (Fig. 3). Shark 365 interacted with other juvenile lemon sharks on 128 occasions with an average of 7 interactions per day, and with up to 6 different individual sharks per day (Fig. 4). For all interactions, over half lasted <30 seconds with about 10% of interactions lasting >3 minutes (Fig. 5). It was possible to assign locations for 10 of these interactions (Fig. 1); half of these were located in the Aya's Spot refuge, an area that has been documented previously as supporting aggregations of juvenile lemon sharks in Bimini, Bahamas [31]. Lemon shark 365 associated with 9 individuals, however interactions with two sharks (#555 and 678) were more common than the other 7, with interactions occurring on 8 and 9 separate days of the 17 day deployment period, respectively (Table 2). In total, shark 365 spent 47 and 45 min interacting with sharks 555 and 678, respectively. For full summary information of all interactions with individual sharks, see Table 2.

**Table 2:** Summary data of shark 365's interactions with other transmitter-tagged lemon sharks, over 14 days.

Shark ID	# Interactions ≥ 60 s	# Interactions <30 s	Total # of Interactions	Total Interaction Time (s)	Longest Interaction (s)	# Days Interacted
249	6	7	13	1140	180	6
344	0	2	2	60	30	2
348	4	7	11	810	240	6
377	2	4	6	300	120	1
456	3	2	5	600	300	4
465	2	2	4	180	60	3
488	9	3	12	1710	420	3
555	17	18	35	2820	240	8
678	16	24	40	2760	360	9
**Total**	**59**	**69**	**128**	**10380**		

The numbers of interactions and individual interaction times were summed for each shark during this time period.

## Discussion

The principal finding of this study is that miniaturised acoustic technology now exists which operates over appropriate spatio-temporal scales to provide useful remotely-retrieved information about group associations in free-ranging sharks. Quantifying group behaviours in sharks is difficult. This is due to the impractical nature of observing animals that are notoriously elusive, large bodied and fast moving [7], [12]. The receivers tested in this study, however, offer a new method for investigating this behaviour, giving temporal information with regard to when an interaction took place and for how long, as well as, spatial information about a possible location for the group, if combined with static receivers (seabed-mounted or surface moored). The captive experiments conducted in this study provide an initial insight into the performance of these receivers. The results from the on-shark trials, in particular, showed detections being regularly logged (at an average of 16.95 times for every 600 seconds) when sharks were close to each other, i.e. within 4 m, and rarely logged (average of <1 detection in 600 seconds) when 10 m apart. In these trials, the five ARX receiver tags performed comparably, however, further deployments are required in order to confirm their consistency and to test statistically for differences between receiver performance. Indeed, numerous questions regarding detection performance remain unanswered. For example, these experiments did not test the ARX receivers in differing water depths or substrate type, which are factors known to affect detection range of other acoustic devices [32]. Also, further trials where sharks are between 5–9 m apart should be trialled to test how regularly transmitter-emitted sound intervals are detected within these distances also.

Proximity receivers may not be suitable for all species or types of research questions. In many studies on fishes the distance between individuals is often used to infer group formation; if individuals are within four body lengths of each other then they are deemed to be in a single group [4], [33]. For the sharks used in this study a maximum detection range of 4 m is equivalent to *ca.* 4 body lengths. The results from this study are therefore really only applicable to marine animals that are similar in body length to the study shark or larger. However, ARX receivers are available with higher or lower detection capabilities, depending on the size of animal that is to be investigated. Further experiments are required in order to test the reliability of such changes to the receiver before deployment on larger or smaller animals. At present, for these units, in order to obtain data the test animal must be recaptured. This means that these receivers are largely applicable to shark species that are small bodied, show high site fidelity and localised movements that will enable recapture and retrieval of the archived data [25], [34]. However, in recent years some shark researchers have used Galvanic-Timed-Release (GTR) mechanisms to recover their data loggers, with a VHF transmitter incorporated to facilitate retrieval [35]. Although still limited to a researcher's operational range at sea, this mechanism could be used for these receivers, making them suitable for use with larger more wide-ranging, albeit coastal, shark species [16].

Although the field trial did not provide enough data to make any firm conclusions about juvenile lemon shark grouping behaviour in general, it did provide some interesting preliminary results enhancing the claim for the technology's future use. The single ARX receiver tag that functioned correctly made contact with 9 out of 15 juvenile lemon sharks, some on multiple occasions (40 contacts with shark 678) and some continuously for up to 7 minutes (shark 488). In combination with an array of seabed-mounted receivers, the locations of a number of these interactions were also identified. With further replicates, this type of information could be used to determine if sharks are grouping or avoiding particular individuals within the population, based on a number of phenotypic traits such as sex, size and species. In recent years social network analysis has been used as a tool to assess animal social group structure using this type of data in other taxa and so could be applicable here also [2], [36].

The formation of lemon shark groups could also be related to abiotic factors such as lunar and tidal cycle, water temperature and depth, as well as biotic factors such as predation risk or prey availability. The ARX receiver tags could be used additionally to monitor much finer scale movement of animals in and out of specific areas, for example caves or refuge sites. It could also prove to be a practical means of quantifying encounter rates between predators and their prey in the marine environment, opening the way for studies of optimal foraging strategies or analysis of predation risk, which have largely eluded detailed study in sharks to date [16], [34]. This technology is also relevant to investigations of reproductive behaviour, such as that seen in nurse sharks [37], where duration and frequency of encounters between refuging females and large numbers of males can be investigated accurately without the need for long-term direct observations. Future trials should look to investigate how the device can deal with multiple sharks interacting and whether the position of an animal within the group affects the number of detections logged, for example whether it is leading or following another individual. In addition, it will be important, at least initially, to combine observations of wild sharks interacting, simultaneously with the deployment of ARX receivers, to allow detections on the devices to be confirmed with actual observations of sharks interacting.

To better understand group living in sharks, previous research has relied primarily on techniques such as captive observations, fishery capture records and position estimates based on triangulation of underwater acoustic receivers [7], [9]–[10], [17]–[18]. New radio-collar proximity detectors in the terrestrial animal literature [20]–[22] prompted the idea and development of a similar type of technology for marine animals. The technique described in this study is a new acoustic proximity method that has demonstrable potential for providing spatial and temporal information regarding the composition and structure of marine animal groups. Although widely recognized in many shark species [5]–[8], [29], [38], grouping behaviour has yet to receive the attention that it has been given in other taxa, such as cetaceans [39], primates [40] and teleost fish [41]. It is widely recognized that groups of marine animals are particularly vulnerable to overfishing whilst in specific locations [42]–[43]. Therefore, understanding their grouping behaviour may provide important information on the types and locations of key habitats and provide spatial foci for species management and conservation, including the placement of marine protected areas. The method described in this study provides an exciting alternative technique for researchers to take advantage of when attempting such studies but, perhaps as importantly, provides a practical means for revealing details of the largely unknown dynamics of shark social behaviours and group structure.
